# Inflammation as a determinant of healing response after coronary stent implantation

**DOI:** 10.1007/s10554-020-02073-3

**Published:** 2021-01-21

**Authors:** Dorota Ochijewicz, Mariusz Tomaniak, Grzegorz Opolski, Janusz Kochman

**Affiliations:** 1st Department of Cardiology, Medical University of Warsaw, Banacha 1a Str., 02-097 Warsaw, Poland

**Keywords:** Inflammation, Healing response, Coronary computed tomography angiography, Optical coherence tomography

## Abstract

Cardiovascular disease remains the leading cause of death and morbidity worldwide. Inflammation plays an important role in the development of atherosclerosis and is associated with adverse clinical outcomes in patients after percutaneous coronary interventions. Data on stent elements that lead to excessive inflammatory response, proper identification of high–risk patients, prevention and treatment targeting residual inflammatory risk are limited. This review aims to present the role of inflammation in the context of evolving stent technologies and appraise the potential imaging modalities in detection of inflammatory response and anti-inflammatory therapies.

## Introduction

Abundant lines of evidence, both from clinical and experimental studies, support the hypothesis that inflammation on top of dyslipidemia has an important role in atherothrombosis [1, 2]. Substantial percentage of patients with atherosclerotic cardiovascular disease with well-controlled low-density lipoprotein cholesterol level and residual inflammatory risk have increased incidence of major adverse cardiac and cerebrovascular accident [3, 4]. Potential molecular link between cholesterol metabolism and inflammation has been recently described through the transcription factor SREBP2 [5]. This suggests the existence of other mechanisms that promote inflammation and new treatment options in this high-risk patient population should be further explored. The Canakinumab Anti-inflammatory Thrombosis Outcomes Study (CANTOS) for the first time provided clinical data showing that targeting inflammation with an antibody against interleukin-1β after myocardial infarction led to a lower incidence of recurrent cardiovascular events than placebo [6]. Available data on healing response after coronary stent implantation in the context of inflammation are scarce. Stent technology evolved over time and different profiles of clinical complications and healing response were observed. Apart from mechanical factors (underexpansion, fracture) local inflammation can lead to aggressive neointimal proliferation, neoatherosclerosis and in consequence in-stent restenosis. [7] This review highlights the role of local and systemic inflammation, possibilities of invasive and non-invasive imaging and potential treatment strategies after coronary stent implantation.

## Local inflammation: should we blame metal, polymer or drug?

**Fig. 1:**
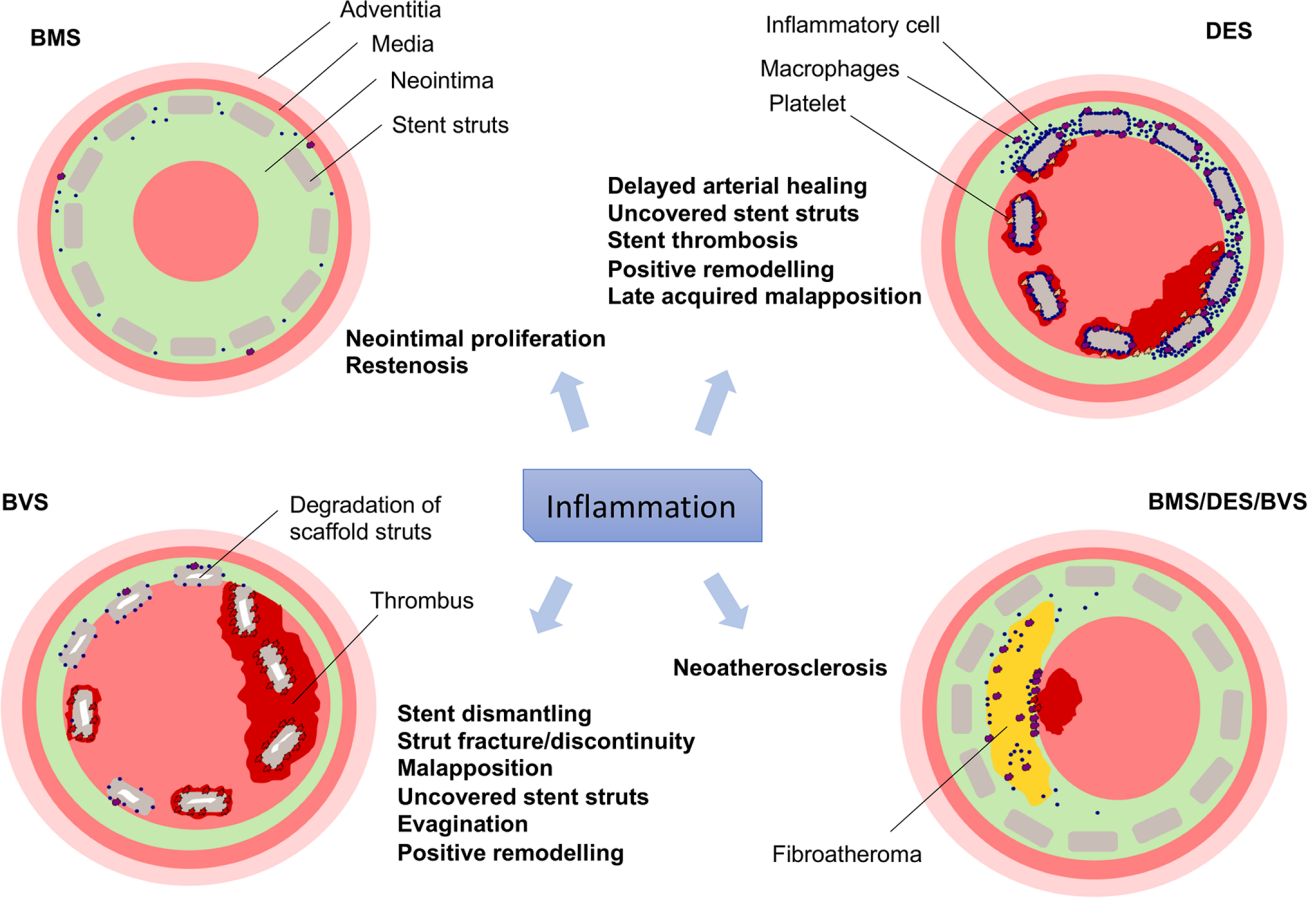
Adverse events potentially related to inflammation after percutaneous coronary intervention. *BMS* bare metal stent, *DES* drug eluting stent, *BVS* bioresorbable scaffold

Theoretically, inflammation can occur against any stent component, including the metal, the antiproliferative agent or the polymer. Autopsy and in vivo imaging studies suggest that chronic long-term inflammation and abnormal vessel healing may contribute to adverse stent-related events [8, 9] (Fig. 1).

## Bare metal stents

Turning point in the history of interventional cardiology—introduction of bare metal stents (BMS) were developed to prevent occlusion and restenosis following balloon angioplasty [10]. After improvement in stent deployment technique and antiplatelet therapy still the risk of neointimal hyperplasia and target lesion revascularization have limited wide utilization of this device [11]. Aberrant vascular smooth muscle cell proliferation and migration is a complex phenomenon with involvement of inflammation after vascular injury [12]. Larger strut thickness have been recognized to impact degree of injury leading to higher inflammation and restenosis [13]. BMS are mostly made from stainless steel, which contains the nickel, chromium, molybdenum and manganese. Although nickel is the most frequent allergen there is no clear evidence that hypersensitivity reaction to metals lead to restenosis after metal stent implantation [14, 15]. However, metal allergy is frequently observed in patients with recurrence of in-stent restenosis [15–17].

## Drug eluting stents

First generation stainless steel sirolimus-eluting stent (SES) and paclitaxel-eluting stent (PES) were able to substantially reduce in-stent restenosis associated with BMS while increasing the incidence of late stent thrombosis (ST) [18]. Autopsy studies reported delay in arterial healing characterized by persistent fibrin deposition, greater inflammatory reaction with signs of hypersensitivity and delayed re-endothelialization when compared with BMS [8, 19, 20]. The most widely accepted mechanism explaining the excess risk of late ST was due to the antiproliferative effect of the drugs released by these devices [8]. Durable polymers used in first generation drug eluting stents (DES): poly(styrene-b-isobutylene-b-styrene) for PES and polyethylene-co-vinyl acetate, and poly n-butyl methacrylate for SES are proposed to be associated with chronic inflammation and eosinophils accumulation [21–24] (Table 1). In the SES polymer releases 80% of the loaded dose of sirolimus in 30 days and the rest by 3 months [25]. The polymer used in PES had a biphasic elution phase of paclitaxel, providing a burst release of 2 days, and subsequently a low-level release over 10 days [26]. In patients with very late stent thrombosis after full release of the drug, histopathological signs of inflammation, delayed-type hypersensitivity reaction and intravascular ultrasound evidence of late acquired malaposition was one of the causes of late ST [20]. It is thought that this hypersensitivity reaction occurs as a result of polymer induced inflammation [21, 27].

**Table 1:** Characteristics of stent type and inflammatory response

Stent name (manufacturer)	BMS	1st DES		2nd DES		BP-DES	BVS
	Bx Velocity (Medtronic)	CYPHER (Johnson & Johnson)	TAXUS Express2 (Boston Scientific)	XIENCE V (Abbott Vascular)	RESOLUTE (Medtronic)	SYNERGY (Boston Scientific)	Absorb BVS (Abbott Vascular)
Drug eluted	–	Sirolimus (1.4 µg/mm^2^)	Paclitaxel (1 µg/mm^2^)	Everolimus (1 µg/mm^2^)	Zotarolimus (1 µg/mm^2^)	Everolimus (1 µg/mm^2^)	Everolimus
Polymer type	–	PEVA and PBMA	SIBS	VDF-HFP and PBMA	C10, C19, and PVP	Bioresorbable PLGA (abluminal coating; bioresorption kinetics: 4 months)	PDLLA
Kinetic of drug elution	–	80% within 30 days; remainder released by the end of 90 days	< 10% at 30 days; 90% remains sequestered within the polymer formulation without further measurable release	80% within 30 days; remainder released by the end of 120 days	70% within 30 days; remainder released by the end of 120 days	50% within 2 months and w100% at 3 months	80% of the drug is released in 28 days
Metal platform	SS	SS	SS	CoCr	CoCr	PtCr	– (PLLA)
Strut thickness [µm)	140	140	132	81	91	74	150
Signs of inflammation	++	+++	+++	+	+	+	++

*PB*-*DES* biodegradable polymer-based drug eluting stent, *C10* polybutyl methacrylate, *C19* polyhexyl methacrylate, polyvinyl acetate, *CoCr* cobalt chromium alloy, *PBMA* poly *n*-butyl methacrylate, *PC* phosphorylcholine, *PEVA* polyethylene-co-vinyl acetate, *PtCr* platinum chromium alloy, *PVDF*-*HFP* polyvinylidene fluoride co-hexafluoropropylene, *PVP* polyvinyl pyrrolidone, *SIBS* poly(styrene-b-isobutylene-b-styrene), *SS* stainless steel, *PLGA* poly(d,l-lactidecoglycolide acid), *PDLLA* poly-d,l-lactide

The introduction of second-generation DES significantly reduced target lesion failure (TLF), especially due to its enhanced safety profile, with lower rates of deaths or major adverse cardiovascular events (MACE) [28, 29]. Histological analysis provide evidence of lower incidence of vascular inflammatory response after second generation everolimus-eluting stents compared with first generation [30]. Thinner stent struts, more biocompatible, durable polymer (composed of vinylidene fluoride and hexafluoropropylene monomers) releasing a reduced dose of drug compared with first-generation DES might be associated with more favorable vascular response [31]. Human autopsy reports showed that the second-generation cobalt-chromium everolimus-eluting stents (CoCr-EES) present lower inflammation response with no hypersensitivity and less fibrin deposition [9]. Efforts to decrease polymer-induced vessel wall inflammation and enhance stent surface endothelialization evolved in development of thinner struts with abluminal bioresorbable polymers so as to modulate directional drug release [32]. In the animal model next-generation bioresorbable polymer DES resulted in lower levels of para-strut inflammation, neointimal foam cell infiltration and neointimal formation at 180 days compared to the permanent polymer DES [33]. Newer-generation DES with thin struts and biodegradable polymers were found to be non-inferior or superior to contemporary durable polymer with respect to composite clinical endpoints [34–36].

The innovation of a special surface morphology for anchoring drugs to the stent-surface to reduce inflammation like polymer-free everolimus-eluting stent (EES) is now under development [37].

## Bioresorbable vascular scaffolds

Permanent nature of metallic struts prevents complete recovery of vascular structure and function with the risk of very late stent failure. Fully bioresorbable vascular scaffold (BVS) were designed to overcome these limitations. The ABSORB BVS (Abbott Vascular) was the most extensively studied device of BVS compared with best in class DES Xience (CoCr-EES). Multiple randomized trials and meta-analyses of the ABSORB trials, showed increased rates of target lesion failure and device thrombosis through 5-year follow up [38–40]. Additionally, differences in the vasomotor reactivity were not observed in the favor of BVS [41]. Accordingly, the device was withdrawn from the market in 2017.

There are several presumed mechanisms of the scaffold failure: a less strong mechanical property of bioresorbable materials, in consequence a larger strut thickness and injury, which can predispose to underexpansion/protrusion/fracture of struts resulting in increased risk of stent thrombosis [42–45]. An ex vivo study has shown greater thrombogenicity and higher inflammation at 28 days after thick strut bioabsorbable EES implantation compared with thin struts biodegradable polymer metallic EES [46]. Histological studies in animal models comparing Absorb BVS and CoCr-EES have shown that inflammation was mild to moderate in Absorb and was greater as compared with DES at 6 to 36 months, although inflammation decreased with time. Both devices exhibited absent or minimal inflammation at 42-month [47]. Histological changes of Absorb dismantling were observed after 12 months with the completed degradation by 36 months [47]. The giant cell infiltration, which is known to be linked to chronic immune responses, has been observed during the resorption process of the scaffolds [48]. However, the prevalence and clinical impact of resorption accompanied by inflammation it is not known.

Studies investigating inflammatory biomarkers: high sensitive C-reactive protein (hsCRP), interleukin-6 (IL-6), tumor necrosis factor (TNF) did not show any systemic inflammatory response after BVS placement [49, 50].

It seems that the results of Absorb trials should not be applicable to other BVS devices due to different mechanical properties of each bioresorbable material (even amongst poly-l-lactic acid) [51]. Comparison of metallic (Magmaris) and polymeric (Absorb) BVS showed that the magnesium scaffold had significantly less platelet adherence, thrombus deposition, and inflammatory cell adherence in ex vivo study 1 h after deployment [52].

## Neoatherosclerosis

Chronic inflammation and impaired endothelial healing with increased lipoproteins migration to the sub-endothelial space contribute to neoatherosclerosis development—one of the mechanism for stent restenosis, late and very late ST [53]. Histologically, it is characterized by accumulation of lipid-laden foamy macrophages within the neointima with or without necrotic core and/or calcification [54]. Neoatherosclerosis occurs earlier and more often in 1st generation DES (31%) compared with BMS (16%) and increases with time in both platforms [54]. The prevalence of neoatherosclerosis in second-generation CoCr-EES (29%) was comparable to the first generation DES [9]. In the short period of time, 18 moths follow up, durable and biodegradable polymer DES showed low percentage of neoatherosclerosis (11.6% vs. 15.9%) [55].

Data regarding neoatherosclerosis after BVS implantation is limited and came from optical coherence tomography (OCT) studies with small number of patients. Moriyama et al. confirmed neoatherosclerosis progression with lumen narrowing in all patients in the in-scaffold segments within 5 years compared with no significant signs of atherosclerotic findings in the out-scaffold segments. Conversely, Karanasos et al. observed favorable neointimal healing with development of a signal-rich, low-attenuating tissue layer [56].

The mechanisms underlying the development of neoatherosclerosis are poorly elucidated. The neoatherosclerosis may occur in months to years following percutaneous coronary intervention (PCI), whereas atherosclerosis in native coronary arteries develops over decades. Stent implantation causes vascular injury and local blood flow disturbances associated with endothelial dysfunction which led to activation of inflammatory cells, increase in thrombogenicity and reduced efflux of β-lipoprotein, which then accumulates within the neointima [57]. Apoptosis of macrophages and smooth muscle cells contribute to the formation of necrotic core and calcification [54]. Immature endothelial cells with increased permeability also promote migration of monocytes [57]. Delayed neointimal healing with incompetent endothelium might promote higher incidence of neoatherosclerosis after 1st generation DES [57]. The underlying native atherosclerotic plaque might as well contribute to the pathogenesis of neoatherosclerosis, however early pathological reports described no anatomical communication with the original atherosclerosis tissue [53].

## Optical coherence tomography: signs of inflammation

**Fig. 2:**
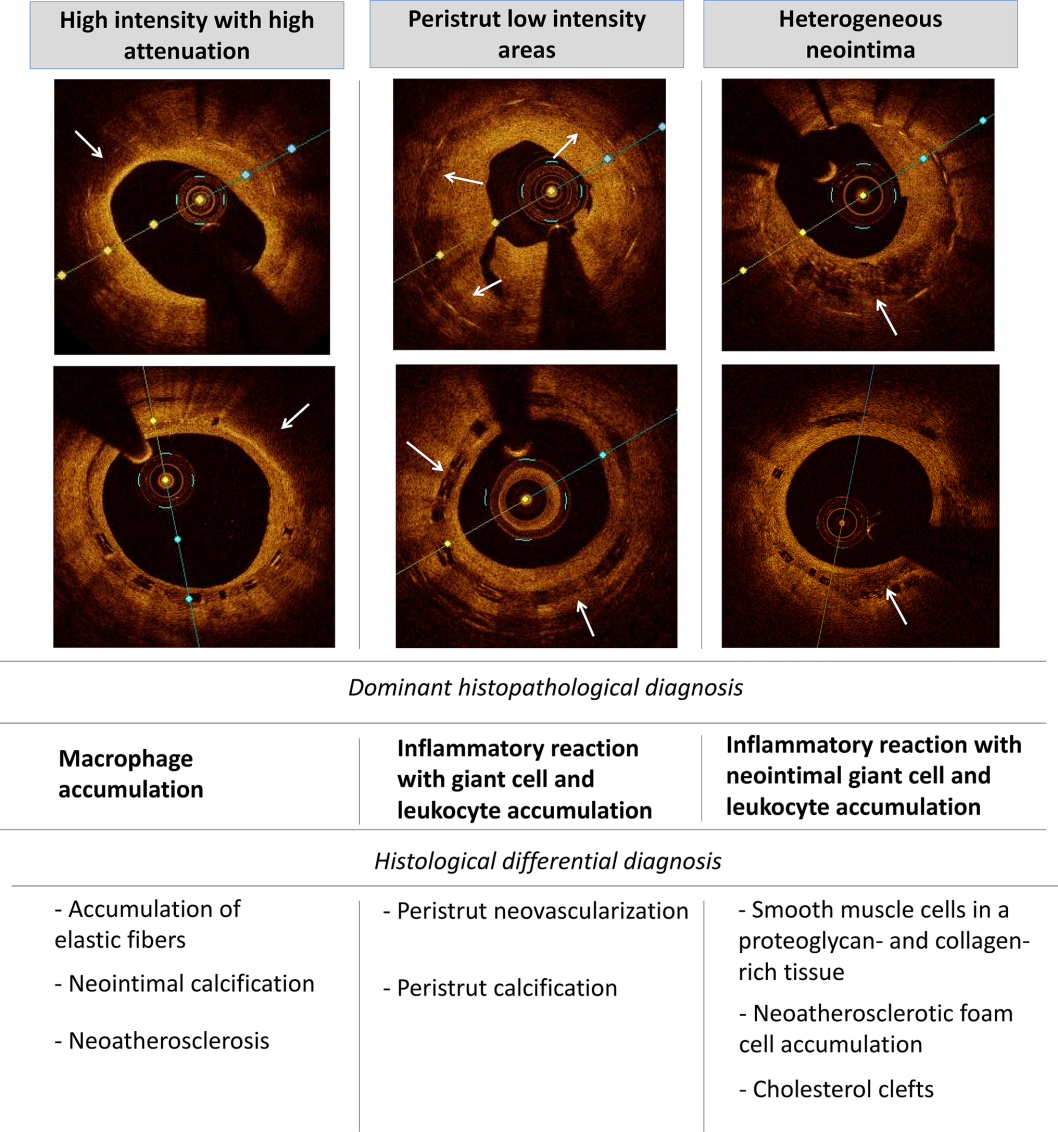
The optical coherence tomography signs of inflammation with the dominant and differential histopathological findings

Among intravascular imaging methods widely used in clinical practice only OCT generates unprecedented intracoronary images with resolution comparable to histological studies. OCT achieved high diagnostic accuracy (90––95%) for the classification of coronary plaques: fibroatheroma, fibrocalcific or fibrous plaque [58]. Still the identification of inflammatory cells is much more challenging. Studies aimed at understanding histological correlations between presumed OCT patterns of inflammation after stenting: heterogenous neointima, peri-strut low intensity areas (PLIA) or high-intensity and high-attenuation pattern suggest average diagnostic accuracy—86%, 30% and 70% respectively [59] (Fig. 2). In heterogenous pattern of neointima histological examination revealed inflammation with neointimal giant cell accumulation (34%), leukocyte accumulation (29%), neoatherosclerotic foam cell accumulation (12%) or cholesterol clefts (11%), healthy neointima (12%), and fibrin accumulation (3%) [59]. PLIA defined as a circumscribed low-intensity area surrounding struts showed a number of different matching histopathological components, including inflammatory reaction characterized by peristrut giant cell accumulation (23%) or peristrut leukocyte accumulation (13%), peristrut neovascularization (36%) and peristrut calcification (18%). High-intensity, high-attenuation pattern revealed a predominance of foam cell accumulation (68%), superficial elastic fibers without foamy macrophages (12%), and neointimal calcification (11%). Despite wide variety of histological differential diagnoses the aforementioned patterns have clinical significance. Observational OCT studies of patients receiving DES showed that presence of heterogenous neointima was linked with major adverse cardiac events over a median 31-month follow-up [60]. The presence of PLIA was associated with an increased rate of target lesion revascularization after everolimus-eluting stent implantation [61]. The foamy macrophage clusters are the earliest feature of neoatherosclerosis [53]. Superficial location of macrophages with co-presence of minimal lumen area < 4 mm^2^ and fibrous cap thickness < 75 μm are validated features of plaque vulnerability [62]. In patients with very late ST macrophage infiltration was more frequent within ruptured plaques whereas calcifications were more common in frames with intact fibrous cap [63]. However, the ability to identify macrophages in optical coherence tomography is limited [64]. Bright spots were correlated with a variety of plaque components that cause sharp changes in the index of refraction (macrophages, cellular fibrous tissue, interfaces between calcium and fibrous tissue, calcium and lipids, fibrous cap and lipid pool) [64]. Additionally, large pools of lipid-rich macrophages corresponded to dark regions [64]. Novel intravascular modality e.g. micro-OCT (mOCT), which offers an axial resolution of 1 μm, may visualize cells more precisely [65].

To explore the issue of proper inflammation cells visualization further investment in technology of greater accuracy must continue. Beyond the morphological characteristics we also need additional physiological features like activity of immune cells, endothelial shear stress, state of endothelial function and hypercoagulation.

## Non-invasive imaging

Clinical applicability of non-invasive imaging modalities in secondary prevention to detect residual inflammatory risk remains limited [66]. Hybrid methods like positron emission tomography/computed tomography (PET/CT) and positron emission tomography/magnetic resonance (PET/MR) co-register PET images with CT or MR anatomical data [67]. 18-fluorodeoxyglucose (18F-FDG) uptake by metabolically active cells (e.g. macrophages) can detect inflammation and assess the efficacy of statin therapy [68]. Although nuclear imaging appears to provide a good solution for the imaging of coronary inflammation, these methods remain expensive, with limited clinical availability and high radiation exposure. Nowadays, the most promising data can be obtained from coronary computed tomography angiography (CCTA). Based on observation that coronary artery inflammation inhibits adipogenesis in adjacent perivascular fat, a novel imaging biomarker—the perivascular fat attenuation index (FAI)—has been proposed to capture coronary inflammation by mapping spatial changes of perivascular fat attenuation [69, 70]. The FAI has excellent sensitivity and specificity for detecting inflammation as assessed by tissue uptake of 18F-FDG in PET [70]. High perivascular FAI values (cutoff ≥ –70,1 HU) were an indicator of increased all-cause and cardiac mortality in two large prospective cohorts of patients undergoing clinically indicated CCTA [69]. FAI could facilitate identification of high-risk individuals, before structural changes of the coronary wall are visible. Additionally CCTA can detect high risk plaque (HRP) features like: the napkin-ring sign, positive remodeling, low attenuation plaque and spotty calcification that are all associated with a high risk of acute cardiovascular events [71]. The combination of HRP and perivascular FAI can better guide the novel therapies for residual inflammatory risk.

## Systemic inflammation

Patients with increased inflammatory status undergoing PCI are at high-risk of adverse clinical outcomes [4, 72, 73]. Both pre- and post-PCI increased C-reactive protein (CRP) and hsCRP level was a prognostic indicator for subsequent cardiac events [4, 74, 75].

Several different pro-inflammatory cytokines such as IL-6, matrix metalloproteinase-9 (MMP-9), and tumor necrosis factor-α (TNF-α) are each associated with coronary heart disease risk independent of conventional risk factors [76]. Even after low density lipoprotein (LDL) cholesterol level reduction by proprotein convertase subtilisin/kexin type 9 (PCSK9) inflammatory status is a strong predictor of adverse clinical results [77, 78]. Statins were also found to have greatest efficacy in the presence of vascular inflammation and reduce CRP level largely independent of LDL reduction [79, 80]. The complex interaction between modest elevations in plasma inflammatory biomarkers, systemic and local factors contributing to development of vulnerable plaques are not yet completely understood. Therefore, it has been hypothesized that targeting different inflammatory pathways might have efficacy in the treatment and prevention of cardiovascular disease.

## Therapies for residual inflammation

The pleiotropic anti-inflammatory and immunomodulatory effect of statins is supposed to provide greater survival benefits in the population with chronic inflammation in addition to its LDL-C lowering effect [80–82]. Statins regulate the functions of T and B lymphocytes, dendritic cells, natural killer cells, reduce the production of inflammatory markers (CRP, TNF, IL-1, IL-6) and reduce the incidence of major cardiovascular events in patients with elevated high-sensitivity CRP levels but without hyperlipidemia [83–85]. Despite intensive lipid-lowering therapy patients with coronary disease and residual inflammatory response remain at high risk for acute cardiovascular events [77]. Various novel anti-inflammatory agents have been investigated in preventing atherosclerotic complications (Table 2). To date, most promising results have been obtained from clinical trials involving canakinumab—targeted interleukin-1ß in patients with previous MI (myocardial infarction) and a high sensitivity CRP ≥ 2 mg/l. The primary endpoint of the study was the first occurrence of myocardial infarction, non-fatal stroke or cardiovascular death. Canakinumab administration have translated to a 15% relative risk reduction for MACE with concomitant increased rates of infection [6]. In the CIRT trial low-dose methotrexate did not reduce cardiovascular events and levels of interleukin-1β, interleukin-6 or CRP than placebo. However the study included all patients with previous MI or multivessel coronary disease regardless of residual inflammatory risk [86]. Recently published COLCOT and LoDoCo2 studies investigated the colchicine—inhibitor of tubulin polymerization, microtubule generation and possibly modifier of molecules adhesion, inflammatory chemokines and the inflammasome. In patients within 30 days after myocardial infarction and in patients with chronic coronary syndrome administration of colchicine significantly decreased ischemic cardiovascular events in comparison to placebo [87, 88]. Given the multifarious nature of inflammation in atherosclerotic processes, there is a clear need for future novel and safe therapies in patients found to have persistent residual inflammatory risk.

**Table 2:** Major randomized clinical trials of anti-inflammatory therapy in cardiovascular disease

Trial	Inclusion criteria	Intervention	Signaling pathway	Size	Results	Benefit observed
Anti-inflammatory Therapy with Cankinumab for Atherosclerosis (CANTOS) [6]	Previous MI and a hs-CRP ≥ 2 mg/l	Canakinumab (subcutaneous injection 50 mg, 150 mg or 300 mg every 3 months) vs placebo	Interleukin-1ß	N = 10,061	HR 0.85; 95% CI 0.74–0.98; p = 0.021 in the 150 mg treated group	Yes
Cardiovascular Inflammation Reduction Trial (CIRT) [86]	Previous MI; MVD with DM2/metabolic syndrome	Methotrexate (15–20 mg weekly) vs placebo	Interleukin-6, TNF	N = 4786	HR 1.01; 95% CI 0.82–1.25; p = 0.91	No
Colchicine Cardiovascular Outcomes Trial (COLCOT) [87]	Previous MI ≤ 30 days	Colchicine (0.5 mg daily) vs placebo	Multiple targets	N = 4745	HR 0.33; 95% CI 0.18–0.59; p < 0.001 HR 0.77; 95% CI 0.61–0.96; p = 0.02	Yes
Low Dose Colchicine (LoDoCo2) [88]	Chronic coronary syndrome	Colchicine (0.5 mg daily) vs placebo	Multiple targets	N = 5522	HR 0.69; 95% CI 0.57–0.83; p < 0.001 HR 0.72; 95% CI 0.57–0.92; p = 0.007	Yes
SOLID-TIMI 52 [89]	Previous MI	Darapladib (160 mg daily) vs placebo	Lipoprotein-Phospholipase A2	N = 13,026	HR 1.00; 95% CI 0.91–1.09; p = 0.93	No
LATITUDE-TIMI 60 [90]	Acute MI	Losmapimod (7.5 mg twice daily) vs placebo	P38 mitogen-activated protein kinases	N = 3503	HR 1.16; 95% CI 0.91–1.47; p = 0.24	No
Aggressive Reduction of Inflammation Stops Events (ARISE) [91]	Previous ACS	Succinobucol (300 mg daily) vs placebo	LDL-Oxidation	N = 6144	HR 1.00, 95% CI 0.89–1.13, p = 0.96	No
VISTA-16 [92]	ACS	Varespladib (500 mg daily) vs placebo	Secretory Phospholipase A2	N = 5000	HR 1.25; 95% CI 0.97–1.61; p = 0.08	No

*ACS* acute coronary syndrome, *HR* hazard ratio, *CI* confidence interval, *LDL* low density lipoprotein, *MI* myocardial infarction, *hsCRP* high-sensitivity C-reactive protein, *MVD* multivessel coronary disease, *DM2* type 2 diabetes mellitus

## Conclusions

Inflammatory responses after percutaneous coronary intervention lead to abnormal neointimal healing and increased risk of adverse clinical outcomes. Neither imaging nor other diagnostic modalities are available to accurately detect inflammation in coronary arteries treated with stent implantation to date. The perivascular fat attenuation index assessed by CCTA appears the most promising non-invasive modality to detect residual inflammatory risk. OCT is most useful intravascular imaging in determining surrogate imaging parameters of inflammation. Residual inflammation, considered as a modifiable pathogenic factor, remains still not adequately addressed for cardiovascular risk modification.
